# Directed ultrafast conformational changes accompany electron transfer in a photolyase as resolved by serial crystallography

**DOI:** 10.1038/s41557-023-01413-9

**Published:** 2024-01-15

**Authors:** Andrea Cellini, Madan Kumar Shankar, Amke Nimmrich, Leigh Anna Hunt, Leonardo Monrroy, Jennifer Mutisya, Antonia Furrer, Emma V. Beale, Melissa Carrillo, Tek Narsingh Malla, Piotr Maj, Lidija Vrhovac, Florian Dworkowski, Claudio Cirelli, Philip J. M. Johnson, Dmitry Ozerov, Emina A. Stojković, Leif Hammarström, Camila Bacellar, Jörg Standfuss, Michał Maj, Marius Schmidt, Tobias Weinert, Janne A. Ihalainen, Weixiao Yuan Wahlgren, Sebastian Westenhoff

**Affiliations:** 1 Department of Chemistry and Molecular Biology, University of Gothenburg, Gothenburg, Sweden; 2 Department of Chemistry – BMC, Uppsala University, Uppsala, Sweden; 3 Department of Chemistry – Ångström Laboratory, Uppsala University, Uppsala, Sweden; 4 Paul Scherrer Institut, Villigen, Switzerland; 5 Physics Department, University of Wisconsin-Milwaukee, Milwaukee, WI USA; 6 Department of Biology, Northeastern Illinois University, Chicago, IL USA; 7 Department of Biological and Environmental Sciences, Nanoscience Center, University of Jyvaskyla, Jyvaskyla, Finland; 8 Present Address: Department of Chemistry, University of Washington, Seattle, WA USA; 9 Present Address: Department of Chemistry and Molecular Biology and the Swedish NMR Centre, University of Gothenburg, Gothenburg, Sweden

**Keywords:** Nanocrystallography, Photobiology

## Abstract

Charge-transfer reactions in proteins are important for life, such as in photolyases which repair DNA, but the role of structural dynamics remains unclear. Here, using femtosecond X-ray crystallography, we report the structural changes that take place while electrons transfer along a chain of four conserved tryptophans in the *Drosophila melanogaster* (6-4) photolyase. At femto- and picosecond delays, photoreduction of the flavin by the first tryptophan causes directed structural responses at a key asparagine, at a conserved salt bridge, and by rearrangements of nearby water molecules. We detect charge-induced structural changes close to the second tryptophan from 1 ps to 20 ps, identifying a nearby methionine as an active participant in the redox chain, and from 20 ps around the fourth tryptophan. The photolyase undergoes highly directed and carefully timed adaptations of its structure. This questions the validity of the linear solvent response approximation in Marcus theory and indicates that evolution has optimized fast protein fluctuations for optimal charge transfer.

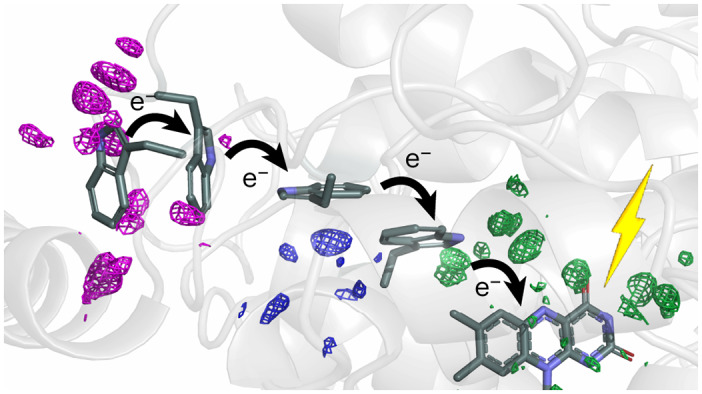

## Abstract (web summary)

Photoinduced electron transfer forms the basis for photosynthesis and DNA repair. Ultrafast structural changes recorded for a DNA-repairing photolyase now reveal specific and directed protein motions accompanying the electron transfer.

## Main

Electron transfer in proteins is important in many biological processes, such as photosynthesis, cellular respiration, oxidative stress defence and denitrification^1–3^. Electron transfer typically occurs between cofactors or amino acid side chains and has been studied for decades^4,5^. A paradigm for electron-transfer reactions in proteins occurs in photolyases/cryptochromes, where a flavin adenine dinucleotide (FAD) cofactor is photoreduced and electrons are donated through a chain of conserved tryptophan residues^6–8^.

Photolyases repair DNA lesions using light as energy source^9^. They are essential for maintaining the integrity of the genome in prokaryotes and many eukaryotes. Photolyases form a protein family together with cryptochromes, which regulate growth and development in plants, entrain the circadian clock to daylight and provide a key component of magnetovision in some animals^10,11^. Despite their diverse functions, photolyases and cryptochromes have remained structurally homologous over billions of years of evolution and have retained their electron-transfer chain. This testifies to the functional importance of photoinduced charge transfer for the protein family.

In both photolyases and cryptochromes, the photoexcited FAD extracts an electron from the nearby tryptophan, Trp407 (numbered for the *Drosophila melanogaster* (6-4) photolyase), within 1 ps, initiating a cascade of electron-transfer reactions along Trp384, Trp330 and Trp381. A long-range (15–18 Å) radical pair between the semiquinone FAD^**·**−^ and the tryptophanyl radical Trp381H^**·**+^ is established^8,12^. In cryptochromes, this is the active signalling state, but in photolyases the photoreduction is repeated a second time, yielding FADH^−^, which is the state that can repair the DNA lesion^13,14^. For stabilization of the semiquinone radical pair, the last tryptophan of the chain typically releases a proton to the surrounding solvent, and the FAD^**·**−^ receives a proton to form FADH^**·**^. Both processes occur on millisecond time scales^12,15^. Electron transfer rates between the tryptophans have been determined^8,12,15^ and more complex reaction pathways have been found, including side reactions to other tryptophans and the adenine group of the FAD^16^.

According to Marcus theory, electron-transfer reactions are controlled by the free-energy gain of the reaction and the ability of the transfer site and environment to stabilize the charge^17^. In photolyases/cryptochromes, transfer occurs along chemically identical tryptophan sites, and therefore free-energy gains must be due to the structure and dynamics of the environment around the sites. This provides evolution with a strong handle to control this process. An interesting situation arises when electron transfer occurs on the same time scale as the environmental relaxation. This leads to non-ergocity and breakdown of the original Marcus theory. This can be expected to be common in proteins in which environmental relaxations stretch over many time scales and take longer than typical electron-transfer events. In these cases, the environmental structural dynamics become very important for the transfer kinetics^18^. Quantum mechanical theories of electron transfer largely confirm this notion^19^, and an approach to cover non-ergocity with a semi-empirical parameter has recently been presented^20^.

Charge transfer during the photoreduction of photolyases has been studied by spectroscopy and simulations^12,15,16,21–25^. Typically, the free-energy gains for each transfer step are a few hundreds of meV and the reorganization energies are moderate (<1 eV). The energies have been estimated from spectroscopically determined electron-transfer rates assuming Marcus behaviour^16^. However, because it is challenging to measure electron-transfer rates between spectroscopically similar tryptophan residues, and since it is questionable if Marcus theory holds, these energies should be considered with some care. The energies and the rates of charge transfer have also been predicted based on molecular simulations^14,21,22^. Although earlier simulations suffered from methodological challenges, such as insufficient sampling of protein conformations or the lack of self-consistent treatments of the induced charges^22,24^, Cailliez et al. have recently reported free energies of the charge transfer of −170 meV, −330 meV and −150 meV and reorganization energies of 400 meV, 750 meV and 600 meV for the three transfer steps along the tryptophan tetrad of a (6-4) photolyase^21^. However, even in this comprehensive study, the energies and rates do not agree with experiments^15^, pointing to a gap in current understanding of electron transfer in photolyases.

A key step forward would be to characterize the structural dynamics associated with charge displacement. The closest information on the subject comes from intraprotein solvation kinetics derived from time-dependent Stokes shift measurements^26,27^. From these experiments it was concluded that water molecules respond to charge density changes within a few picoseconds, that combined water and protein movements take tens of picoseconds, and that large-scale breathing motions of the protein lead to solvation dynamics at hundreds of picoseconds. However, these data are not structure specific, and it remains poorly understood how protein structural changes guide electron-transfer reactions.

Femtosecond time-resolved serial crystallography (SX) opens up the possibility of resolving protein structural changes upon photoexcitation^28,29^. The technique has been used to decipher structural mechanisms in photosynthetic, sensor and transport proteins^30–35^. With respect to photolyases, a nanosecond time-resolved SX study has been carried out for the two reduction steps on a *cis*–*syn* cyclobutane pyrimidine dimer (CPD) photolyase^36^, and we have recorded a crystallographic snapshot of the end state of the first photoreduction in a (6-4) photolyase at 300 ms (ref. ^37^). Here we capture structural snapshots for the (6-4) photolyase from *Drosophila melanogaster* (*Dm*(6-4) photolyase) covering the earliest femto- and picosecond time scales. We identify distinct protein structural changes which accompany the electron transfer at the FAD and along the tetrad of tryptophans, visualizing how highly directed movements of amino acids and water molecules accompany electron-transfer reactions in photolyases.

## Results

### Substantial difference electron density observed

**Fig. 1:**
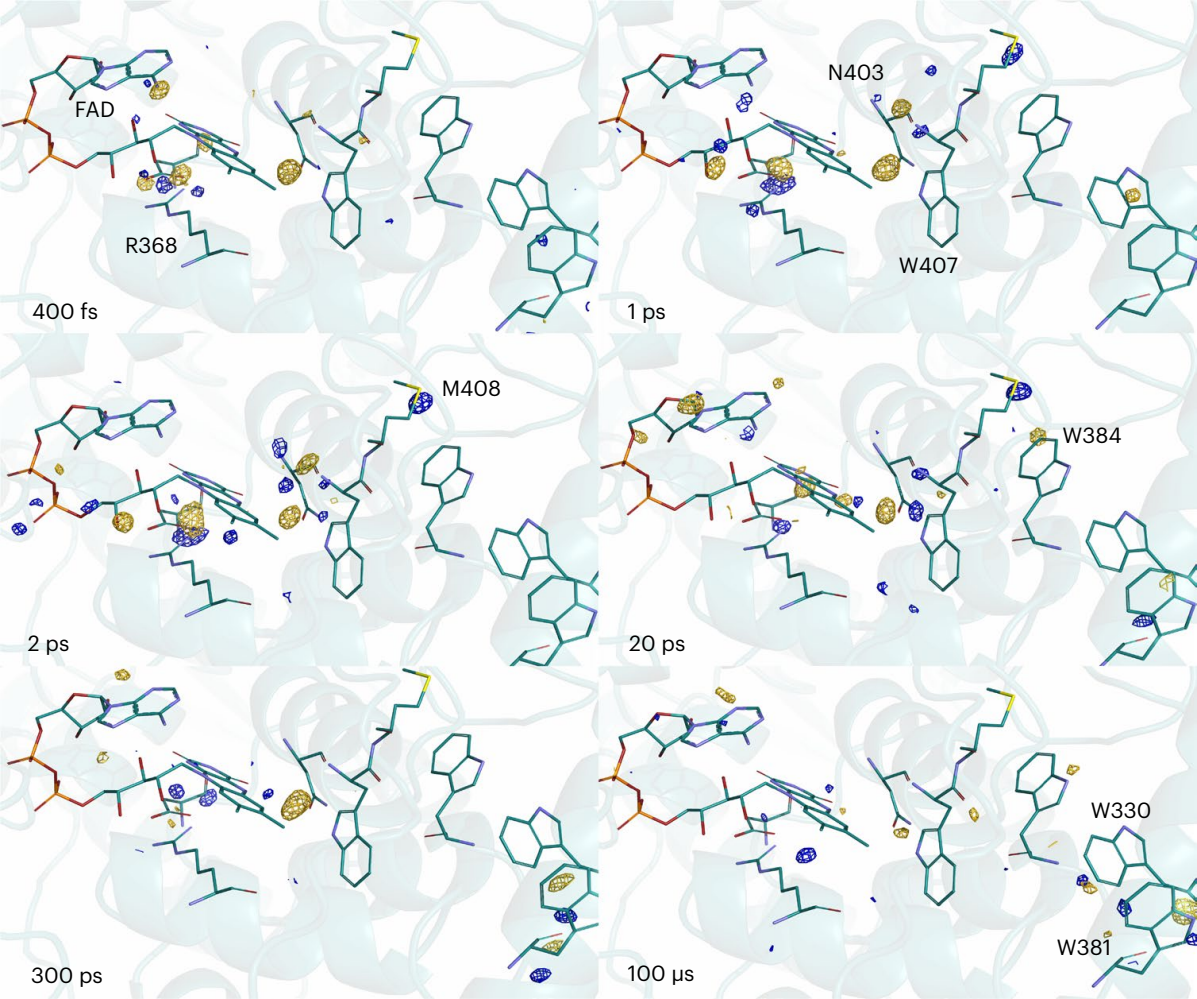
Time-resolved structural changes along the tetrade of tryptohans observed. Observed DED obtained at 400 fs, 1 ps, 2 ps, 20 ps, 300 ps and 100 μs after light activation. The maps are contoured at 4*σ*. The dark structure is shown in all panels in cartoon representation with the FAD and key residues as sticks. The colours of the sticks correspond to different atoms: C, cyan; N, blue; O, red; P, orange; and S, yellow. Negative and positive features are depicted in gold and blue, respectively.

**Extended Data Fig. 1:**
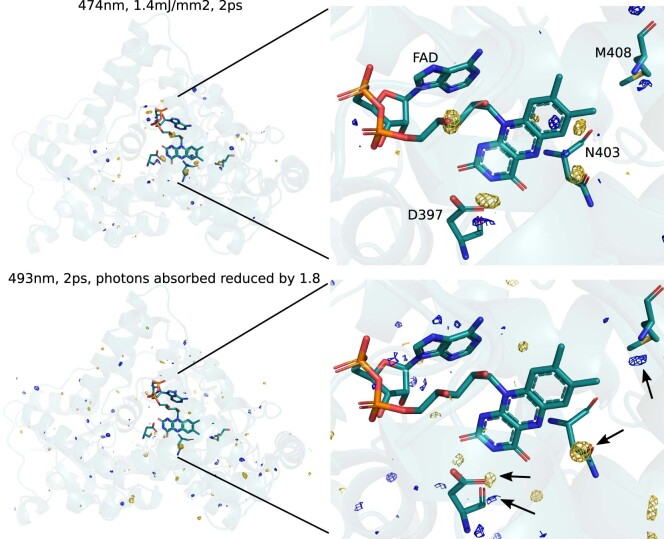
Lowering the photon flux by a factor 1.8 reduced the DED signal. The upper panels show the DED presented in the main paper at 2 ps (photoexcitation at 474 nm, 1.4 mJ/mm^2^). The lower panels show a DED map recorded at 2 ps after photoexcitation at 493 nm (2.9 mJ/mm^2^). The maps are contoured at 0.012 eÅ^−3^ (upper) and 0.014 eÅ^−3^ (lower) respectively. Because 493 nm is at the red onset of absorption, the number of photons absorbed at 493 nm is expected to be lowered by a factor of 3 compared to excitation at 474 nm (assuming a Gaussian spectrum of the laser). Thus, the number of absorbed photons is expected to be 1.8 times lower (2.9/3 mJ/mm^2^ for the experiment at 493 nm compared to 1.4 mJ/mm^2^ for the experiment at 474 nm), consistent with the observed lower DED signal amplitude. In a third measurement at even lower excitation fluence (1.1/3 mJ/mm^2^ at 493 nm), we did not find any DED signals above noise. We note that the extinction coefficient of the FAD is low (approx. 10000 M^−1^ cm^−1^ at *λ* max) compared to for example rhodopins approx. 60000 M^−1^ cm^−1^), where similar or higher excitation fluences were used in SX experiments^32^. Thus, we conclude that the excitation fluence of 1.4 mJ/mm^2^ at 474 nm used for all measurements in the main paper was within the one photon regime.

First, we were curious to find out if the structural changes associated with electron transfer in photolyases can be determined by time-resolved SX. We recorded time-dependent crystallographic data from microcrystals of *Dm*(6-4) photolyase dispersed in a cellulose matrix. Photoexcitation was performed by an optical laser pulse with a pulse length of 150 fs, with a centre wavelength of 474 nm and at a fluence of 1.4 mJ mm^−^^2^. We observed difference electron density (DED) features above noise for all time points from femto- to microseconds (Fig. 1). Lowering the effective photon fluence by a factor of 1.8 reduced the signal, and lowering it by a factor of 8 made it vanish (Extended Data Fig. 1). We therefore conclude that the excitation fluence is in the regime of one-photon absorption.

**Fig. 2:**
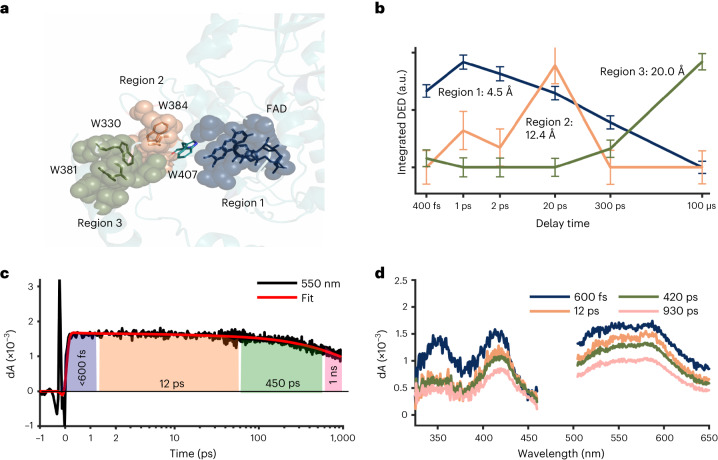
Observed changes in electron density correlate with the kinetics of the electron-transfer reactions. **a**, The residues for three regions grouped around the first, second and third/fourth tryptophan are indicated as spheres of different colours. Region 1: FAD, R368, D397, N403; region 2: W384, E385, M408, S411, S413; region 3: I328, P329, W330, T376, W381, N490. **b**, The integrated DED is displayed for the three regions as a function of delay time. Integration was over a radius of 1 Å around the atoms of each region and for DED above 3.1*σ* of the map. The plot for each region is normalized at its maximum. The error bars indicate the noise floor of the DED map from which the data point was extracted, as estimated by the average of the integrated signal (>3.1*σ*) over 5,000 randomly selected regions in the map, each consisting of six residues, excluding the residues in regions 1–3. The error bars are scaled with the same normalization factor as was used for the intensities for regions 1–3. **c**, Kinetic trace of transient absorption data at 550 nm with the time constants derived from global analysis of the transient absorption data indicated as shaded regions. The fit is a representative fit of the kinetics from the global analysis. **d**, Transient absorption spectra at selected pump–probe delays after excitation at 470 nm. Parts **b** and **c** were prepared with MATLAB R2021a. Source data

To characterize how the DED evolves with time, we integrated the DED in each map for three regions of residues (Fig. 2b), which are defined in Fig. 2a. We found that the kinetics for the three regions are distinct: region 1 is close to the chromophore (the average distance of the Cα atoms of the residues from N5 of the FAD was 4.5 Å) and the integrated DED peaks first at 1 ps; region 2 around the second tryptophan, Trp384 (12.4 Å distance from N5), tops at 20 ps; and region 3 around the fourth tryptophan, Trp381 (20.0 Å from N5), rises at late time points (300 ps and 100 μs). The observed DED changes are rather small and indicate time-dependent, concerted structural changes of a few residues around key charge-transfer sites (Fig. 1).

**Extended Data Fig. 2:**
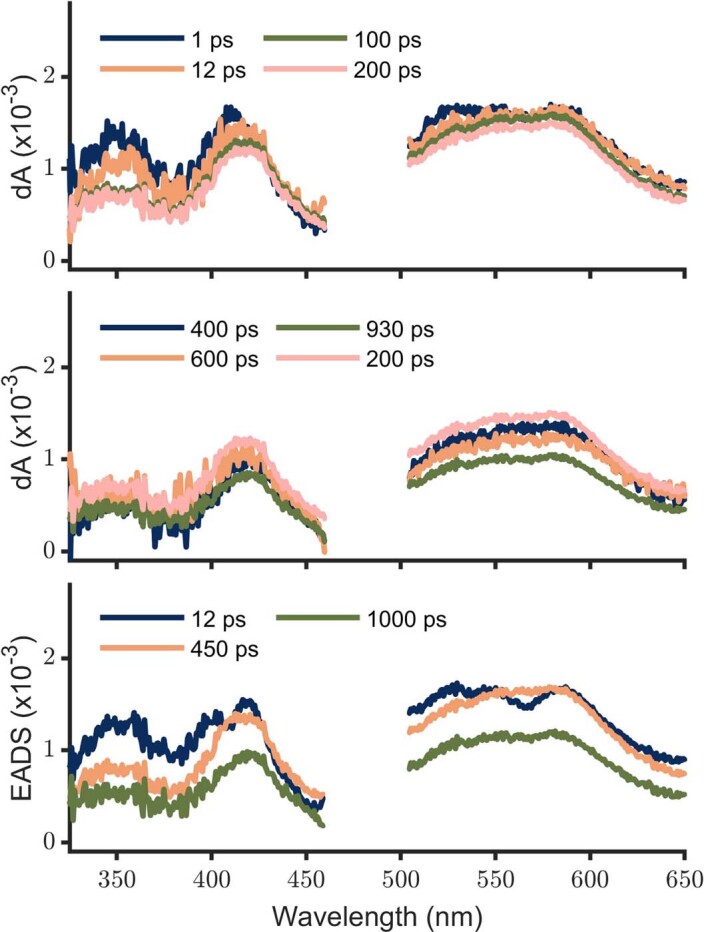
Overview of femtosecond transient absorption spectra. Top and middle: Transient absorption spectra at selected pump–probe delays after excitation at 470 nm and the same conditions as described in the main paper. Bottom: EADS spectra generated after global analysis of the transient absorption data with a sum of 4 exponentials in Glotoran. The first EADS (decay time of 400 fs) is not shown since it was convoluted with coherent artefacts. The decay time of the last EADS component is close to the end of the time range and has therefore a large uncertainty, it reflects long-lived charges, which outlive the detection window of 1 ns. Source data

To further characterize the charge-transfer kinetics, we recorded femtosecond transient absorption data for *Dm*(6-4) photolyase (Fig. 2c,d). Based on the spectral shapes, a spectral decomposition into four spectral components (Extended Data Fig. 2) and comparison with the literature^12,15,23,38^, we find that charges are separated between the FAD and Trp407 within 520 fs, that charges transfer between tryptophans with characteristic time constants of 12 ps and 450 ps, and that charges live longer than the detection window. The fast charge-separation time (<520 fs) agrees well with our observations from time-resolved SX, where we observe a substantial DED signal at 400 fs in region 1 (Fig. 2b). The two picosecond components (Fig. 2c) cannot be assigned to a specific charge-transfer process based on the spectral shapes alone. However, their characteristic times overlap with the rise and decay of the DED peak of region 2 (Fig. 2b) and we therefore assign them to the charge transfer to and from the second tryptophan, Trp384. The spectroscopy does not conclusively resolve the transfer between the third and fourth tryptophan. Nevertheless, the crystallographic data indicate that the charges reach the final tryptophan within 300 ps, indicating that the transfer from the third to the fourth tryptophan is so fast that it cannot be observed in the spectra. From the spectroscopy and crystallography data together we conclude that the DED reveals protein structural changes which occur close to the electron-transfer sites and follow the flow of electrons through the protein.

### Ultrafast stabilization of the photoinduced FAD radical

**Fig. 3:**
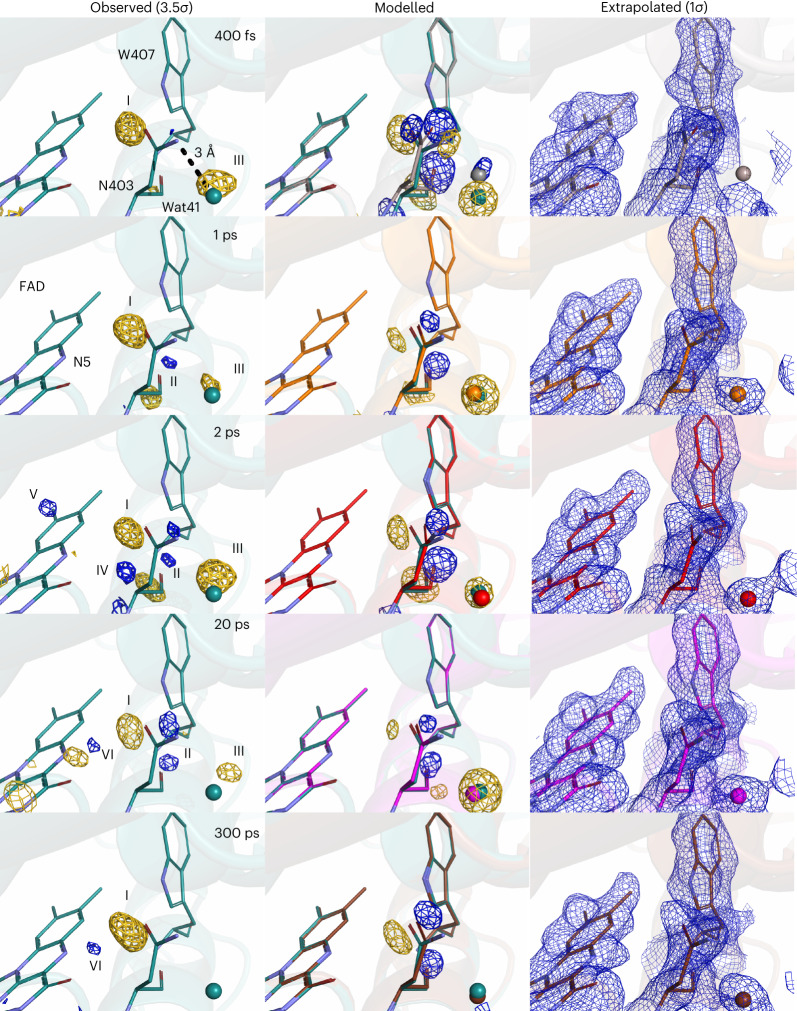
Conformational changes around Asn403. The dark structure (cyan) and the observed DED maps are shown in the left-hand column. The light structures superimposed with the dark structure and the calculated DED maps are shown in the middle column. The negative and the positive features are depicted in gold and blue, respectively. The light structures and the 2*F*_e_ − *F*_c_ electron-density maps (blue) of FAD, asparagine 403 (N403) and the surrounding water molecule Wat41 are shown in the right-hand column.

**Extended Data Fig. 3:**
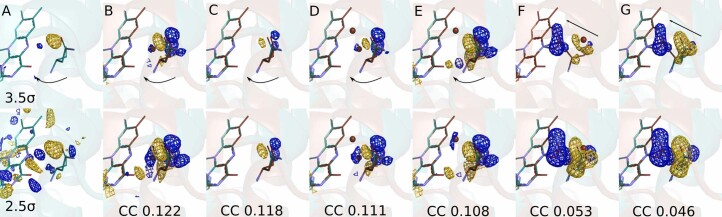
Alternative models for the positioning of Asn403 do not lead to good agreement between observed (A) and calculated (B-G) difference electron density at 300 ps. Each map is shown at two different sigma levels (upper and lower row). We modelled and computed calculated difference map of Asn403 with two water molecules (D), one water molecule at different positions (B and E) and without water molecule (C). The Pearson Correlation Coefficient (CC) values change only moderately between models B-E and we therefore present the simplest model (without water, C) in the main paper. Alternative to the models with water, we moved the Asn403 closer to the FAD (G) and placed a water nearby (F). The experimental DED is not reproduced and the CC drops drastically for the models E and F. The Pearson Correlation Coefficient (CC) values calculated between the observed and calculated DED within a sphere of 7 Å radius centred on Asn403 are shown. Gold indicates negative and blue positive DED.

Next, we focus on the DED features observed around the FAD chromophore. Close to N5 of FAD and the first tryptophan (Trp407) of the tetrad, we observe strong DED features on asparagine 403 (Asn403) during (400 fs) and following (1 ps, 2 ps, 20 ps and 300 ps) charge separation (Fig. 3, features I and II). Residues at the position of Asn403 are known to be crucial for stabilization of the charge on the FAD in photolyases/cryptochromes. To visualize the time dependence of the DED features, we integrated the DED over a radius of 2 Å around Asn403 (Fig. 5b)^39^. The kinetics of Asn403 show an instantaneous signal, a further rise up to 20 ps and a decay for later time points. We refined Asn403 using real-space refinement against extrapolated maps (third column in Fig. 3), using visual agreement between experimental (first column in Fig. 3) and calculated DED maps (second column in Fig. 3) to ensure good fits. From 400 fs to 20 ps the side chain of Asn403 twists, so that the carbonyl group moves away from the N5 of FAD (Table 1). Water 41 (Wat41), which is within hydrogen-bonding distance of the amino group of Asn403 and faces away from FAD in the dark structure, follows this movement (Fig. 3, feature III). We attempted several other movements of Asn403, including a different rotamer, or moving the carbonyl group much closer to the N5 of FAD into hydrogen-bonding distance, but these alternative models did not lead to good agreement between the observed and calculated DED (Extended Data Fig. 3). We conclude that the side chain of Asn403 reacts to the change in electrostatics when FAD becomes reduced. However, it does not undergo a change big enough to stabilize the FAD^**·**−^ through a direct hydrogen bond.

**Table 1:** Time-dependent distances (Å) between various residues

Delay time	Asn403(C=O)–FAD(N5)	Asn403(NH_2_)–FAD(N5)	Asn403–Trp407	Asp397(OD1)–Arg368(NH1)	Asp397(OD2)–Arg368(NH2)
Dark	3.66	5.24	2.93	2.98	2.75
400 fs	4.59	4.86	2.98	3.18	2.84
1 ps	3.90	5.46	3.06	3.33	2.67
2 ps	4.21	5.23	2.87	3.20	2.70
20 ps	3.87	5.21	2.88	3.12	2.65
300 ps	4.06	5.45	2.90	3.06	2.46

### Water dynamics around FAD

In addition to the movement of Asn403 itself, we observe several positive DED features between the Asn403 and the N5 of FAD. These are most pronounced (>3.5*σ*) at 20 ps and 300 ps (Fig 3, feature VI) and also presented at 2 ps albeit with different shapes and in different positions (features IV and V). We attribute these DED features to water molecules which occupy the space between Asn403 and the N5, thereby hydrogen bonding to the negatively charged N5 and stabilizing the charge on the FAD^**·**−^ on picosecond time scales.

The kinetics of these positive DED features are interesting (Fig. 5b). At time delays of 400 fs and 1 ps no discernible positive DED features are observed, features IV and V are only present transiently at 2 ps, and feature VI arises after this time point. The water response in the chromophore-binding pocket is delayed with respect to the response of the side chain of Asn403 and the charge-transfer time. It is dynamic in time and space, indicating that the water molecules traverse through a series of states to reach their final positions.

### Ultrafast response of the Asp397–Arg368 salt bridge

**Fig. 4:**
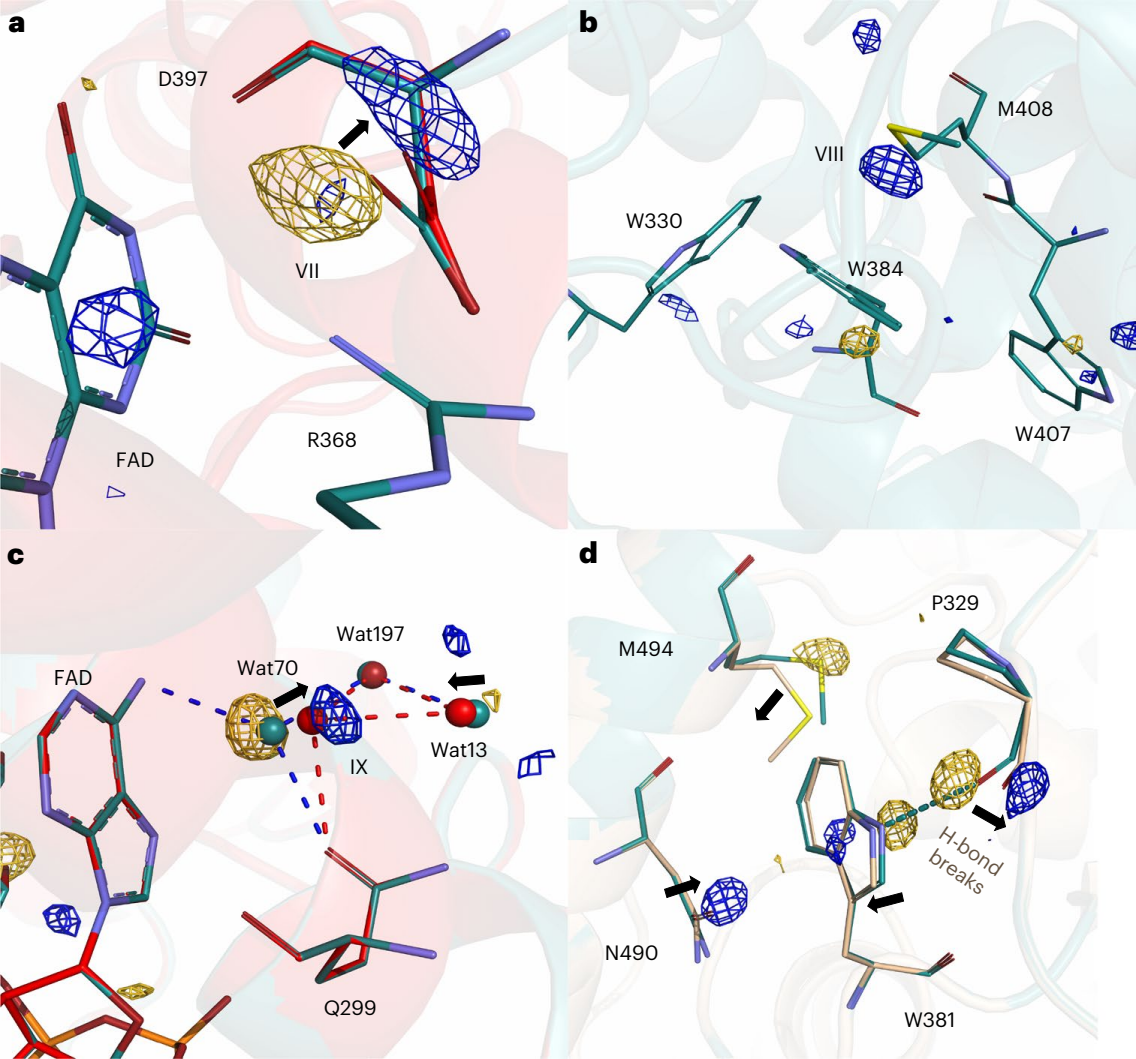
Femto- to microsecond conformational changes around key residues. **a**, Observed DED at 2 ps and 3.5*σ* with the dark structure (cyan) superimposed on the light structure (red) obtained at 2 ps. **b**, Observed DED map around Met408 at 20 ps is displayed together with the dark structure (cyan), contoured at 3.5*σ*. **c**, Observed DED at 2 ps and 3.5*σ* around the adenine moiety of FAD, with the dark structure (cyan) superimposed on the light structure (red). Hydrogen-bonding networks of FAD, Gln299 and water molecules Wat70, Wat197 and Wat13 are shown as blue dashed lines in the dark structure and red in the light structure. **d**, Observed DED map and superimposed dark (cyan) and light (yellow) models around the final tryptophan, Trp381, at 100 μs. The hydrogen bond between the backbone of Pro329 and Trp381 in the dark structure is shown as a dashed line (blue). In all maps, the negative and the positive features are depicted in gold and blue, respectively. Arrows indicate movement of atoms upon photoexcitation.

**Fig. 5:**
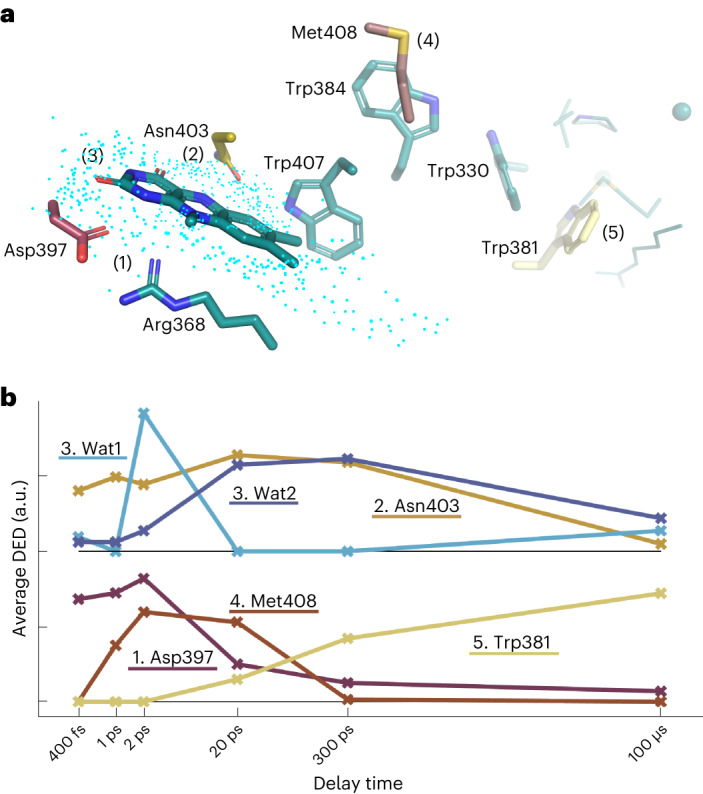
Summary of photochemical events. **a**, Key residues and processes. (1) FAD is reduced and Asp397 and Arg368 respond immediately. (2) Asn403 reacts similarly fast and undergoes a slow phase of response up to 20 ps. (3) A delayed (from 1 ps) and complex motion of water molecules is completed at 20 ps. (4) Met408 undergoes a photoreaction from 1 ps to 20 ps. (5) Trp381 is oxidized at 300 ps, with structural changes evolving around it up to 100 μs. **b**, The kinetics of the observed DED at key positions are shown. For water features the electron density is averaged over positive DED >2*σ* and for the amino acids over negative DED <2*σ* (side chains only). The radius of integration was 2.5 Å. Wat1 corresponds to feature V and Wat2 to feature VI in Fig. 3. The kinetics for water and Asn403 are vertically offset. Source data

Next, we turn our attention to the conserved salt bridge between Asp397 and Arg368, which is located on the opposing side of the FAD compared to Asn403. Figure 4a illustrates a movement of the side chain of Asp397 through the correlated positive and negative feature VII, which is present at 400 fs, maximal at 1 ps and 2 ps, and muted at 20 ps and which has decayed below noise by 300 ps (Fig. 5b and Supplementary Video 1). Refined light structures indicate a transient distance increase across the salt bridge caused by rotation of Asp397 (Table 1). Since the response of the salt bridge is present at 400 fs and decays after 2 ps, we conclude that it is an effect of the sudden change of electrostatics on the reduced FAD. We did not detect a light-induced interaction between Arg368 and FAD as was observed on microsecond time scales^36^. We tentatively attribute this difference to the fact that FAD reoxidizes in the photolyase crystals, and that this occurs faster for the *Dm*(6-4) photolyase compared with the *Methanosarcina mazei* CPD photolyase studied in ref. ^36^.

### Picosecond conformational changes of the FAD chromophore

On FAD itself, we do not detect strong signals that can be attributed to the bending of the isoalloxazine ring of FAD. We observe some negative features on the pyrophosphate chain at 1 ps and 2 ps, but the meaning of those signals is not clear. At 2 ps, 20 ps and 300 ps we find correlated positive and negative DED close to a cluster of water molecules (Fig. 4c, feature IX). We attribute this to a signature of transient oxidation of the adenine through electron transfer to the isoalloxazine ring of FAD. This side reaction has been observed spectroscopically on picosecond time scales^12^. The refined positions of the water molecules show that the hydrogen bond between the adenine and water 70 (Wat70), which is present in the dark, is broken in this process (Fig. 4c).

### Protein response along the tryptophan tetrad

**Extended Data Fig. 4:**
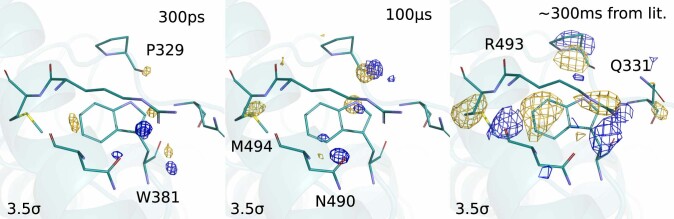
Comparison of DED at 300 ps and 100 *μ*s delay times to literature data. Observed difference electron density maps at 300 ps and 100 *μ*s around the last tryptophan (Trp381). The electron densities were compared to previously published data^37^.

We now turn our attention to changes along the tryptophan tetrad. As in the previous SX experiments on photolyases^36,37^, we did not observe DED signals on the tryptophan side chains themselves above 3.5*σ*, except for signals on the fourth tryptophan (Trp381) from 20 ps (Fig. 1 and Extended Data Fig. 4). In particular and related to the first electron-transfer step from Trp407 to FAD, which occurs within 470 fs (Fig. 2c), we did not observe changes in the reciprocal distance either between FAD and Trp407 or between Asn403 and Trp407 (Table 1). This indicates that the protein environment of Trp407 is very rigid, or that the second tryptophan donates its electron extremely quickly to Trp407.

From 1 ps up to 20 ps, we observe a strong positive signal next to the sulfur atom of the conserved methionine (M408) (Fig. 4b, feature VIII). The feature has a delayed rise (maximum at 2 ps) and decays to zero amplitude by 300 ps (Fig. 5b and Supplementary Movie 1). The sulfur atom of Met408 is located only 4.1 Å from the side chain of Trp384. The signal consists of a positive DED (Fig. 4b), which can be explained by the dynamic localization of an atom in the positive density. We propose that this change is due to a water molecule that is located close to the methionine sulfur of Met408 and transiently oxidizes it.

Finally, the data reveal prominent DED features at the fourth tryptophan (Trp381) for the late time point (100 μs) (Fig. 4d and Extended Data Fig. 4), with a signal present already at 20 ps and 300 ps (Figs. 1 and 5b). We interpret these changes to be due to the arrival of the charge at the last tryptophan Trp381. At 100 μs, we detect structural changes at Trp381, Pro329, Asn490 and Met494, indicating that the hydrogen bond between Pro329 and Trp381 in the dark state breaks, that the side chain of Trp381 moves, and that Asn490 is moving closer to Trp381. The DED around Trp381 is weaker than what we detected previously at 100 ms, but features appear at similar positions (Extended Data Fig. 4)^37^. Based on this and consistent with the expected electron arrival time^12,15^, we assign Trp381 to be in its cationic radical state (TrpH^**·**+^), before deprotonation to the solvent occurs. The signal on the final trpytophan at 100 μs also serves as an internal reference for the present measurements: existence of the signal implies that the photoexcitation yielded in the crystal is notable and that the DED follows the expected flow of electrons through the protein.

## Discussion

Photolyases harvest solar energy through a two-step photoreduction process that then enables them to repair DNA lesions. The protein thereby balances two partially opposing objectives. Charge recombination has to be minimized for maximal collection efficiency, which is achieved by swift separation of charges into a long-range radical pair. At the same time, the charges have to remain accessible for repairing DNA lesions. This implies that the radicals should not be trapped in deep energy minima, which would render the subsequent processes less effective. These are the same requirements as in photosynthetic proteins, where photogenerated charges have to be transported to the opposite side of the membrane.

In photolyases and cryptochromes, the electrons transfer between chemically similar tryptophan side chains. The free-energy gain per transfer step is relatively low at a few hundreds of meV, depending on the transfer step^12,16,21,22,25^. The reorganziation energy is only marginally higher (<1 eV) for the forward reactions^12,25^. This indicates that the charge transfer is driven by the structure and dynamics of the environment of the transfer sites.

Here, we discover a series of timed and specific structural responses upon charge transfer, which we summarize in Fig. 5 and Supplementary Movie 1. The highly conserved salt bridge Asp397–Arg368 shows the fastest response to photoreduction of FAD (event 1 in Fig. 5 and Supplementary Movie 1). It is present from 400 fs onwards and decays until 300 ps. The second fastest response is for Asn403 (event 2 in Fig. 5 and Supplementary Movie 1). Asn403 is conserved among CPD and (6-4) photolyases, and substituted into an aspartate in plant cryptochrome, and a cysteine in *Dm* cryptochrome. The residue was found to form a hydrogen bond to FAD in its semiquinone state^36,37^. Here, we establish that Asn403 does not reach direct hydrogen-bonding interaction with the N5 of FAD on picosecond time scales, contrary to what could have been envisaged^40^. Third, water molecules localize close to the N5, probably to hydrogen bond to it (event 3 in Fig. 5). The time scale of the water solvation is consistent with expectations^41^. While the decay of water feature VI (Fig. 3) follows the decay kinetics of Asn403 and FAD^**·**−^, the build-up of the water signals is delayed with respect to the signals on Asn403 and is more complex, involving different sites around the N5. Accordingly, water position 1 in event 3 (also shown in Fig. 3, feature V) is populated first at 2 ps, and water 2 (Fig. 3, feature VI) takes over afterwards (Fig. 5b).

These three events, which stabilize the charge on the FAD^**·**−^, are not only coordinated in space, but also in time. The temporal response is more complex than expected, with the response of Asp397 decaying faster than the expected lifetime of the FAD^**·**−^ and the structural responses of the waters and Asn403 on the opposing side of the chromophore (Fig. 5b). The side-chain movements are somewhat faster than that of the waters, which is complex in itself (see above). Once the new water network is established around the FAD, the side chain of Asp397 can relax. This is reminiscent of the compensatory movements of the corresponding residues in a CPD photolyase, which were observed on microsecond time scales by time-resolved SX^36^.

**Extended Data Fig. 5:**
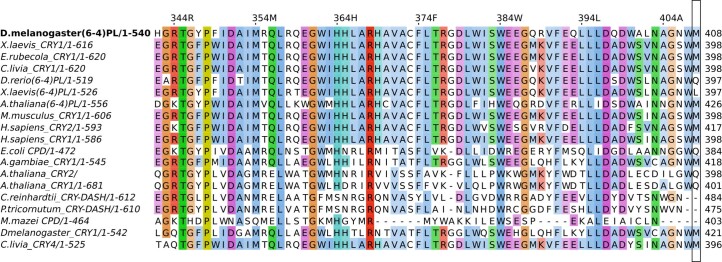
Sequence alignments of some members of (6-4)photolyase, animal and plant cryptochromes and bifunctional CRY-DASH. The sequence alignment reveals that Met408 is conserved among animal cryptochromes. The alignment was performed in Jalview 2.11.1.4 with Clustal using the default settings. Source data

Further down the charge-transport chain, Met408 is of special interest (event 4 in Fig. 5 and Supplementary Movie 1). Met408 is conserved among animal photolyases, and in the wider family it may be replaced by a glutamine (Extended Data Fig. 5). The photoreaction of Met408 is timed from 1 ps to 20 ps by our measurement. One possibility is that Met408 participates directly in the electron-transfer chain as an transient electron donor^42^, or that its oxidation is a by-product of the transient charge that resides on the nearby Trp384. Met408 does not appear to be an active element in the photolyase charge-transfer chain. A methionine has recently been implicated in the stabilization of charged FAD embedded in a light-oxygen-voltage domain^43^ and has been discussed as a possible residue for stabilization of electron transfer in a molecular dynamics study on a (6-4) photolyase^21^. We suggest that the response facilitates long-range charge separation. Met408 seems ideal for this because it is located next to the second tryptophan, which does not have many water molecules close by, and methionines can be reversibly oxidized.

Moreover, the appearance of the signal on Met408 within 1 ps indicates a surprisingly fast charge-transfer time, both with respect to our own spectroscopic characterization of the protein (Fig. 2d and Extended Data Fig. 2) and the literature^15^. The fast transfer can be rationalized if ultrafast coherent charge delocalization is considered. In this model, the photogenerated charge density becomes distributed over a few charge-transfer sites almost instantaneously after photoexcitation, which is possible if delocalized, high-energy molecular orbitals are accessed. This is reminiscent of what has been demonstrated for charge transfer in organic photovoltaic materials^44,45^ and may be in line with a recent proposal for photosynthetic reaction centre proteins^46^. Such a coherent mechanism would help tremendously in suppressing unwanted charge recombination.

At the end of the electron-transfer chain the final tryptophan, Trp381, shows a signal starting from 20 ps (Fig. 5b). Although this appears to be consistent with spectroscopic results for the *Xenopus laevis* (6-4) photolyase, which suggested that the fourth electron transfer take places within ∼40 ps (ref. ^47^), even this response is surprisingly fast and may indicate the influence of coherent transfer. At 20 ps and 300 ps the signal appears directly on the tryptophan side chain and at 100 μs it has spread to nearby residues, demonstrating a larger structural response than the other sites (event 5 in Fig. 5 and Supplementary Movie 1), similar to what has been observed for a CPD photolyase and by us for the present (6-4) photolyase at 100 ms (refs. ^36,37^). Apparently, the protein sequence and structure around Trp381 provide plasticity and movements can occur, whereas at the other sites, the protein scaffold is much more rigid. This illustrates how evolution has optimized the protein sequence around the charge-transfer sites to achieve efficient charge separation.

Up to now, the structural photoresponses of photolyases have been characterized by time-resolved SX on nano- to microsecond time scales^36^, and on the millisecond time scale after photoexcitation^37^. The present work adds the femtosecond to picosecond response to this. A common focal point in all studies is Asn403 and the Asp397–Arg368 salt bridge. In this work, we establish that the two sites react in coordinated ways around FAD on picosecond time scales, which is reminiscent of their response on the microsecond time scale^36^. The structural response of the tryptophans itself is mute (this work), but substantial changes are observed around the final tryptophan (this work and ref. ^37^).

To conclude, we report the ultrafast structural changes that occur in a eukaryotic (6-4) photolyase after photoreduction of FAD to FAD^**·**−^. Crystallographic snapshots on femto- to picosecond time scales reveal distinct protein conformational rearrangements, where side chains and water movements act in concert and accompany charge separation. We propose that these features have evolved to stabilize the radical pairs to provide for efficient charge generation and to avoid recombination, but not to trap the charges too deeply in one of the sites. Most conformational changes of the protein environment are driven by changes in electrostatics. We find that the events are timed with respect to each other, which suggests that the proteins have not only evolved for optimal positioning and electrostatics of the charge-transfer sites, but that evolution has also selected for optimal picosecond dynamics. Our data suggest surprisingly fast charge transfer as detected at Met408 within 1 ps and at the last tryptophan in 20 ps, which may be an indication for ultrafast coherent charge transfer. The findings presented in this paper should be critically considered when estimating charge-transfer rates by Marcus theory. The structural dynamics presented here provide a showcase for the active and highly specific involvement of the protein matrix in electron-transfer reactions, which we envisage to be not only important for DNA repair, but also for the many other enzymatic processes that rely on charge transfer.

## Methods

### Protein expression

Protein expression and purification were performed as described in ref. ^37^, with some modifications. A pET21d+ plasmid containing a codon-optimized gene for *Dm*(6-4) photolyase was used and transformed in BL21(DE3) *Escherichia coli* strain for expression (see Source data for plasmid and protein sequence). The gene and amino acid sequences of the plasmid and protein are given below. The cells were grown in a Studier-like, ZYP-5052-rich medium for autoinduction (for 1 litre: 929 ml ZY (10 g tryptone, 5 g yeast extract), 1 ml 1 M MgSO_4_, 20 ml 50x5052 (0.5% glycerol, 0.05% glucose, 0.2% α-lactose), 50 ml 20 × NPS (1 × NPS: 200 mM PO_4_, 25 mM SO_4_, 50 mM NH_4_, 100 mM Na, 50 mM K), 500 μl of 100 mg ml^−1^ carbenicillin). The cells were grown at 37 °C until the OD_600_ was equal to 0.1, then the flasks were moved to 20 °C for 16–18 h. Cells (15–20 g) were resuspended in 100 ml of buffer A (100 mM Tris–HCl pH 7.6, 50 mM NaCl, 1 mM EDTA, 5 mM dithiothreitol, 5% glycerol), which was supplemented with two tablets of EDTA-free protease inhibitor (cOmplete Protease Inhibitor Cocktail, Roche), and then sonicated on ice at 30% amplitude for 10 min with 10 s pulse on and 30 s pulse off. The supernatant was collected after 30–40 min of centrifugation at 100,000*g* (70 Ti rotor, Beckman) and loaded on a heparin column (HiTrap Heparin HP 5 ml, Cytiva), washed with buffer A, eluted with a linear gradient from 0% to 100% buffer B (100 mM Tris–HCl pH 7.6, 800 mM NaCl, 1 mM EDTA, 5 mM dithiothreitol, 5% glycerol). Fraction 7 (Extended Data Fig. 7) was concentrated and injected via a capillary loop into a size-exclusion chromatography column (HiLoad Superdex 200 pg 16/600, Cytiva) and eluted with buffer A. Fraction 10 (Extended Data Fig. 7) was concentrated with a Vivaspin 20 centrifugal concentrator (molecular weight cut-off, 30 kDa, Sartorius). The concentration of the protein was determined from the absorption at 450 nm (NanoDrop 1000 ultraviolet–visible spectrophotometer, Thermo Scientific) using 11,300 M^−1^ cm^−1^ as the extinction coefficient. Ultraviolet–visible absoprtion spectra were recorded in the dark and light to establish photoactivity. Illumination was for tens of seconds with a light-emitting diode lamp at 455 nm at approximately 116 mW cm^−2^ and the reactive fractions of the proteins were employed for crystallization (Extended Data Fig. 8).

### Crystallization

For the preparation of microcrystals, we first grew macrocrystals by the vapour-diffusion technique, crushed these macrocrystals and used them as seeds for growing microcrystals. Macrocrystals were prepared in hanging drop plates. The drops (2 μl) consisted of 1 μl of protein solution (15–20 mg ml^−1^) and 1 μl of reservoir solution (0.1 M Bis-Tris, pH 6.5, 0.2 M lithium sulfate monohydrate, 22% PEG 3350 and 0.5% ethyl acetate). Macrocrystals grew for 2 days. For the preparation of 50 μl of the seed stock, crystals from six drops were first crushed with a pipette tip and resuspended in a solution made of 19 μl of buffer A and 19 μl of reservoir solution. Next, the crystals were further crushed with seed beads from Hampton Research (HR2-320) by vortexing for several minutes. Samples of seeds were checked under a microscope (Nikon SMZ18) after each vortexing run to evaluate the quality of the seeds. The size range of the macrocrystals was 850–950 μm × 5–9 μm. These crystals were crushed in 1-min steps, four consecutive times, yielding crystals of around 25–30 μm × 4–5 μm in the first step, 15–20 μm × 4–5 μm in the second step, around 15 μm × 4 μm in the third step and finally around 8 μm × 4 μm in the fourth step. We used a cell counter (Hausser Scientific) to estimate the number of seeds to 8 × 10^6^ seeds per ml; however, this includes only seeds with a size >4 μm × 4 μm because smaller ones were difficult to observe with certainty under the microscope. Each microcrystal batch was made by mixing 20 μl of the seed stock, 40 μl of reservoir solution and 40 μl protein solution (15–20 mg ml^−1^) at 4^∘^C. Microcrystals grew after 2 days at 4 °C to a concentration of 5 × 10^7^ crystals per ml (Extended Data Fig. 9, left panel).

### Embedding microcrystals into carrier medium

Hydroxyethyl cellulose (HEC) medium was prepared at a concentration of 22% (w/w) in MQ water (18.2 MΩ·cm at 25 °C). The crystals were spun down at 2,000*g* for 3 min and the supernatant was removed. The microcrystal slurries were mixed into the HEC medium in a ratio 1:3 (HEC/crystal) with a system comprising of three coupled Hamilton syringes^48^. To avoid crystal damage the mixing was done with 2–3 strokes. A microscope image of the crystals after mixing with HEC shows that there is no visible sign of damage from the mixing procedure (Extended Data Fig. 9, right panel), and further evidence of limited crystal damage is provided by the high diffraction quality of the crystals upon X-ray exposure (1.8 Å resolution for the dark structure).

### Data acquisition

Data were collected at the Alvra instrument at the SwissFEL. X-ray pulses with a photon energy of 12.06 keV, a pulse energy of 455–510 μJ and a pulse duration of 50 fs (r.m.s.d.) and at a repetition rate of 100 Hz were used for the experiment. The diffraction images were recorded on the Jungfrau 4M detector with gain switching and in single-pixel autogain mode^49^. We used the viscous injector available at the beamline^48^. The microcrystals dispersed in 22% HEC were extruded at 5 μm min^−1^ from a capillary with an inner diameter of 75 μm. The jet had approximately the same diameter as the capillary. For optical excitation a laser pulse of 150 fs duration at a centre wavelength of 474 ± 12.5 nm, with a total energy of 9.6 μJ in a focal spot of 80 × 86.4 μm^2^ (1/e^2^) beam, was used. The laser fluence was 1.4 mJ mm^−2^. A dark dataset was collected and afterwards datasets with delays of 400 fs, 1 ps, 2 ps, 20 ps, 300 ps and 100 μs in respect to a 474 nm laser were recorded. We recorded 42,547 indexable frames for the dark, 21,576 for the 400 fs time delay, 55,898 for 1 ps, 52,708 for 2 ps, 98,625 for 20 ps, 31,979 for 300 ps and 31,505 for 100 μs.

### Data processing and analysis

Peak finding and indexing were performed with CrystFEL with the command “–indexing=xgandalf –peaks=peakfinder8 –threshold=4000 – –int-radius=2,3,6 –min-snr=3.5 –min-peaks=8 –min-pix-count=2 –min-res=800 –tolerance=10,10,10,8 –median-filter=3”^50^. The stream files were then used as input to Ambigator with “–y 4/m –w 4/mmm –highres=1.7 –lowres=10 –iterations=10”. Later, the Ambigator output was fed to Partialator for scaling and postrefinement processes. We used xsphere as model for treating partialities. The hkl reflection files were then converted to a mtz file and the mtz file from each time point was used for calculation of the DED maps.

The DED maps are the real-space representation of the difference structure factor amplitudes Δ*F*_o_ = *w*(*F*_o_(light) − *F*_o_(dark)) and phases from the dark model^33^. The weighting factor (*w*) was determined for each reflection and down-weights large-amplitude spatial frequencies at low resolution and amplitudes, which are associated with large experimental errors^51^

1 $$w={\left(1+\frac{{(\Delta F_{\mathrm{o}})}^{2}}{\left\langle {(\Delta F_{\mathrm{o}})}^{2}\right\rangle }+\frac{{({\sigma }_{\Delta F_{\mathrm{o}}})}^{2}}{\left\langle {({\sigma }_{\Delta F_{\mathrm{o}}})}^{2}\right\rangle }\right)}^{-1}.$$

DED maps were calculated with 16 Å and 1.9 Å as a low- and high-resolution cut-off, respectively, and the *F*_o_(light) and *F*_o_(dark) were scaled using scalit of ccp4 to match each other and to match the scale of *F*_c_(dark), which is the calculated structure factor from a model refined against *F*_o_(dark).

We employed PHENIX 1.19.2-4158 for the refinement of dark structures against *F*_o_(dark)^52^. Before each refinement run, manual adjustments were carried out in coot^53^. We used the model 3CVY for molecular replacement in PHENIX. To generate models of the photoinduced structural changes, we used real-space refinement in COOT against maps computed from 2*F*_e_ − *F*_c_. *F*_e_ is the amplitude of the extrapolated structure factor, which represents the structure of the pure photoexcited species, and was estimated as^32,54^

2 $$F_{\mathrm{e}}=F_{\mathrm{c}}({\mathrm{dark}})+{N}_{\mathrm{e}}\Delta F_{\mathrm{o}}.$$

**Extended Data Fig. 6:**
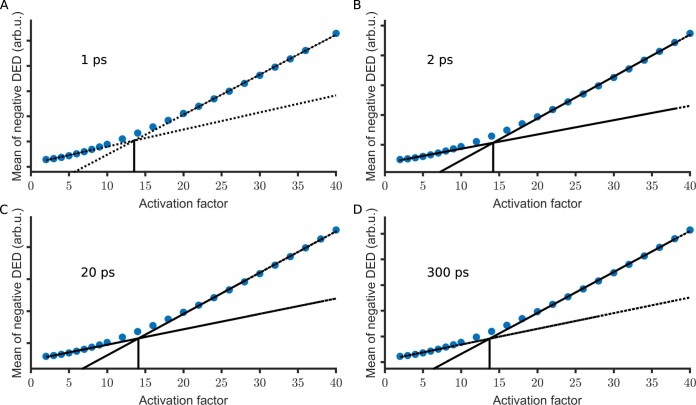
Determination of photoactivation yield. Mean negative DED features (<-2*σ*) plotted as function of the activation factor at 1 ps (A), 20 ps (B), 300 ps (C) and 100 *μ*s (D). The point of intersection in the extrapolated (dashed lines) region of linear fits (solid line) determines the level of activation. Source data

*N*_e_ is the extrapolation factor, which is related to the photoactivation level (*r*) by *N*_e_ ≈ 2/*r*. The factor 2 arises from the difference Fourier approximation and implies that difference maps computed from Δ*F*_o_ only have half of the expected signal strength, even if *F*_o_(light) and *F*_o_(dark) have been scaled to absolute units, as was done here (see above)^55^. We estimated *N*_e_ ≈ 14 from analysis of the negative densities in *F*_e_ computed as a function of *N*_e_ (Extended Data Fig. 6).

After refinement, the accuracy of the structural models was evaluated by comparison between the real-space maps of observed Δ*F*_o_ and calculated $\Delta{F}_{\mathrm{c}}=F_{\mathrm{c}}^{\;\mathrm{light}}-F_{\mathrm{c}}^{\;\mathrm{dark}}$ structure factors. We also computed the Pearson correlation coefficient (CC) in real space as CC = cov(map1,map2)/(*σ*_map1_ × *σ*_map2_), where *σ* denotes the standard deviation of the map. Increasing CC values indicate better agreement. The crystallographic statistics are reported in Extended Data Table 1.

### Femtosecond transient absorption spectroscopy

Transient absorption experiments were carried out using a titanium/sapphire-based amplifier with integrated oscillator and pump lasers. The laser fundamental (800 nm, 3 kHz) was split into pump and probe by a beam splitter, which were directed toward the sample chamber (TAS, Newport). The pump beam at 470 nm was generated by using an optical parametric amplifier (TOPAS NirUVis, Light Conversion). The continuum probe beam was generated by focusing a few mJ per pulse of the amplifier fundamental onto a translating CaF_2_ crystal. Prior to the sample cell, the pump was passed through a depolarizer and attenuated using a neutral-density filter. The probe path was controlled by an optical delay (<3 ns), allowing the transient spectra at varying pump–probe delay times to be recorded on a silicon diode array (Newport, custom-made). The energy used to excite the samples was set to 450 nJ per pulse, focused on a surface of roughly 8,000 μm^2^ (estimated using a Thorlabs beam profiler at the sample position). The fluence of the pump laser was about 30% less than the levels previously reported for transient absorption experiments on a photolyase, which were within the linear photoexcitation regime^47^. Our own power-dependent measurements, although not presented here, provide further confirmation of this. The pump and probe beams were focused onto the sample placed between two CaF_2_ windows separated with a 1 mm spacer. The window containing the sample was mounted on a translation stage and moved continuously in the vertical direction to refresh sample volume and avoid sample degradation. The sample window was kept at approximately 5 °C by continuously pumping water from a refrigerated circulator bath through a flow inlet. A constant flow of nitrogen on both sides of the sample window was maintained throughout the experiments to avoid condensation. The transient absorption data were initially processed using SurfaceXplorer software from Ultrafast Systems, implementing corrections for both the dispersion of the probe beam and any shifts in the time-zero point. The data were then globally fitted to a sum of exponential functions, each convoluted with the instrument response function Gaussian. Each dataset represents an average of five independent scans, which were analysed individually for any inconsistencies that might indicate photodegradation. The presented results correspond to a dataset for which no photodegradation was observed throughout the entire experiment. The global analysis of the data and calculation of the evolution-associated decay spectra were performed using the R package TIMP and its graphical user interface Glotaran.

### Reporting summary

Further information on research design is available in the Nature Portfolio Reporting Summary linked to this article.

## Online content

Any methods, additional references, Nature Portfolio reporting summaries, source data, extended data, supplementary information, acknowledgements, peer review information; details of author contributions and competing interests; and statements of data and code availability are available at 10.1038/s41557-023-01413-9.

### Supplementary information

Reporting Summary

Supplementary Video Movie summarizing the structural changes reported in the paper.

### Source data

Source Data Fig. 2B Data used for plotting electron density in 1D.

Source Data Fig. 2C,D Femtosecond transient absorption data source.

Source Data Fig. 5 Data used for plotting electron density in 1D.

Source Data Extended Data Fig. 2 Data used for plotting transient absorption spectra at selected pump–probe delays.

Source Data Extended Data Fig. 5 Sequences used for alignment.

Source Data Extended Data Fig. 5 Protein and plasmid sequence used for the alignment and for protein expression.

Source Data Extended Data Fig. 6 Data used for the activation level determinations.

**Extended Data Fig. 7:**
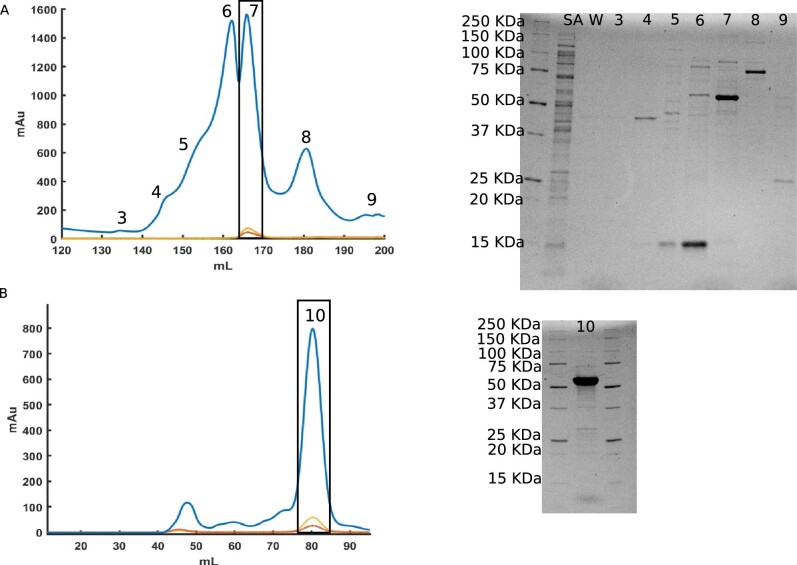
Chromatograms and SDS PAGE gels after Heparin column purification (A) and size exclusion column (B). Fraction 7 is the only fraction with absorbance at 445 nm and 390 nm indicating the presence of the flavin chromophore in the fraction. Fraction 7 was injected in the size exclusion column. In the chromatograms, the blue line indicates absorbance at 280 nm, the yellow line absorbance at 445 nm and the red line absorbance at 390 nm. On the SDS PAGE gel, SA corresponds to the sample application flow through and W to the wash with buffer A before the elution step. Both chromatograms are from 1 out of at least 25 purifications, in which similar chromatograms were recorded. Source data

Source Data Extended Data Fig. 7 Uncropped gel photograph.

Source Data Extended Data Fig. 7 Data for the chromatograms in Extended Data Fig. 7.

**Extended Data Fig. 8:**
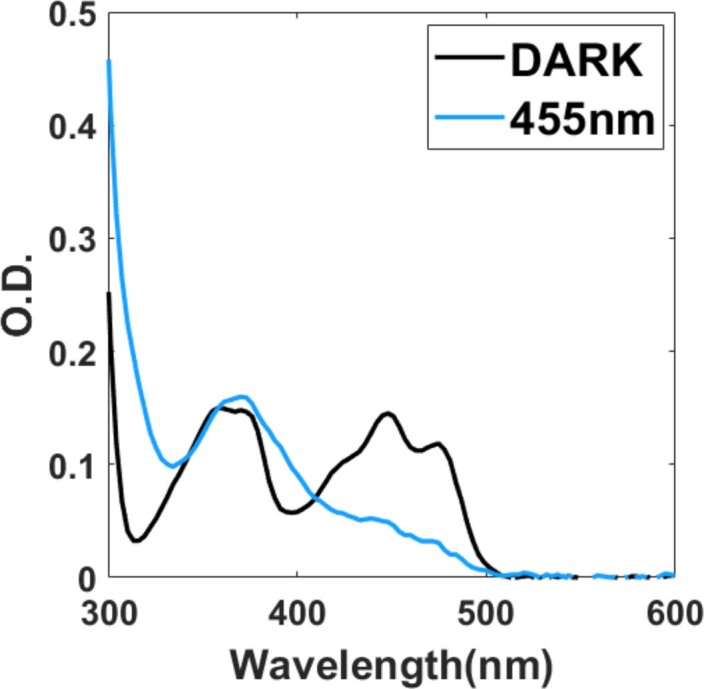
UV-Vis absorption spectra. Absorption spectra of *Dm*(6-4)photolyase before (black plot) and after (light blue plot) illumination at 455 nm is shown. See methods and materials for more details. The spectra shift is characteristic for photoreduction of the FAD chromophore. Source data

**Extended Data Fig. 9:**
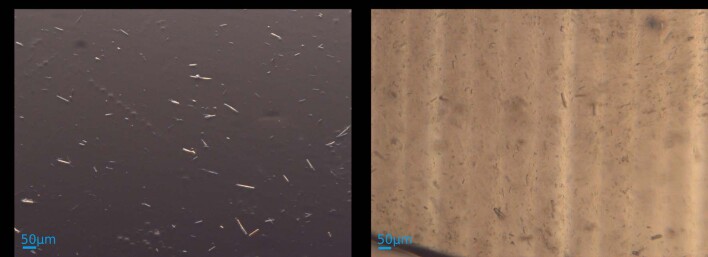
Microscope image of Dm(6-4)photolyase microcrystals after crystallization. The crystals were needle-like with a varying length and a width of <4 *μ*m (left panel). Microscope image of *Dm*(6-4)photolyase microcrystals dispersed in HEC carrier medium (right panel). The micrographs are from 1 out of at least 25 batches of microcrystals, which were used in the beamtime.

Source Data Extended Data Fig. 8 Data corresponding to the ultraviolet–visible spectra.

## Figures and Tables

**Extended Data Table 1:**
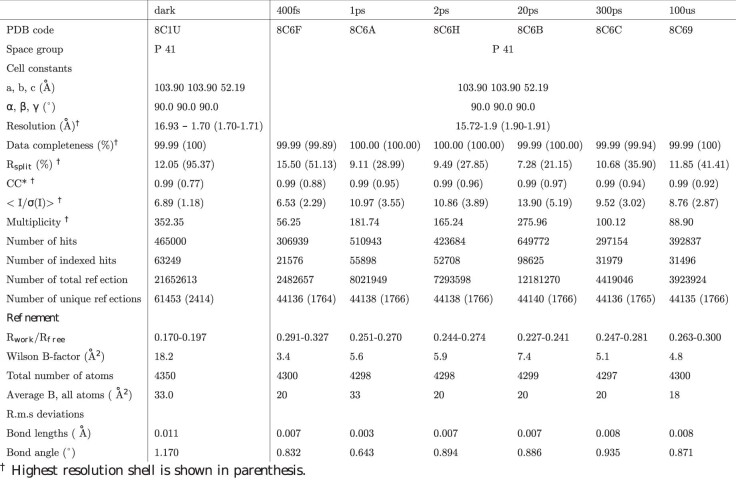
Data and refinement statistics

## Data Availability

The SX data generated are available through the CXIDB database with accession code ID 219. The structural models will be made available through the Protein Data Bank with accession codes 8C1U, 8C6F, 8C6A, 8C6H, 8C6B, 8C6C and 8C69. Source data are provided with this paper.
