# Supplementary figures and images for: Directed ultrafast conformational changes accompany electron transfer in a photolyase as resolved by serial crystallography
Source: Nat Chem. 2024 Jan 15;16(4):624–32. doi: 10.1038/s41557-023-01413-9 (PMC10997514; doi:10.1038/s41557-023-01413-9)

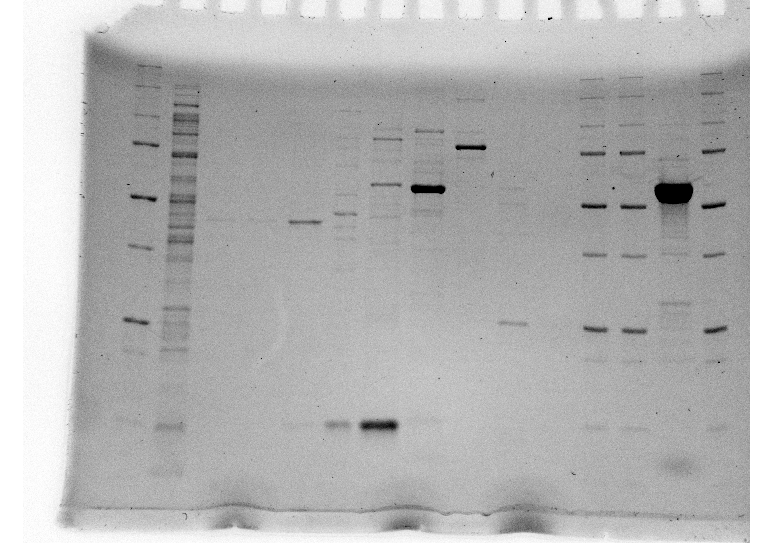

Supplement: Supplementary file 10 — Uncropped gel photograph. [file 41557_2023_1413_MOESM10_ESM.tif]
